# Differences Among Incidence Rates of Invasive Listeriosis in the U.S. FoodNet Population by Age, Sex, Race/Ethnicity, and Pregnancy Status, 2008–2016

**DOI:** 10.1089/fpd.2018.2548

**Published:** 2019-04-10

**Authors:** Aurelie M. Pohl, Régis Pouillot, Michael C. Bazaco, Beverly J. Wolpert, Jessica M. Healy, Beau B. Bruce, Mark E. Laughlin, Jennifer C. Hunter, John R. Dunn, Sharon Hurd, Jemma V. Rowlands, Amy Saupe, Duc J. Vugia, Jane M. Van Doren

**Affiliations:** ^1^ Center for Food Safety and Applied Nutrition, U.S. Food and Drug Administration, College Park, Maryland.; ^2^ National Center for Emerging and Zoonotic Infectious Diseases, Centers for Disease Control and Prevention, Atlanta, Georgia.; ^3^ Tennessee Department of Health, Nasvhille, Tennessee.; ^4^ Connecticut Emerging Infections Program, Yale University School of Public Health, New Haven, Connecticut.; ^5^ New York State Department of Health, Albany, New York.; ^6^ Minnesota Department of Health, Saint Paul, Minnesota.; ^7^ California Department of Public Health, Sacramento, California.

**Keywords:** listeriosis, foodborne disease epidemiology, *Listeria monocytogenes*, foodborne illness

## Abstract

*Listeria monocytogenes* is a foodborne pathogen that disproportionally affects pregnant females, older adults, and immunocompromised individuals. Using U.S. Foodborne Diseases Active Surveillance Network (FoodNet) surveillance data, we examined listeriosis incidence rates and rate ratios (RRs) by age, sex, race/ethnicity, and pregnancy status across three periods from 2008 to 2016, as recent incidence trends in U.S. subgroups had not been evaluated. The invasive listeriosis annual incidence rate per 100,000 for 2008–2016 was 0.28 cases among the general population (excluding pregnant females), and 3.73 cases among pregnant females. For adults ≥70 years, the annual incidence rate per 100,000 was 1.33 cases. No significant change in estimated listeriosis incidence was found over the 2008–2016 period, except for a small, but significantly lower pregnancy-associated rate in 2011–2013 when compared with 2008–2010. Among the nonpregnancy-associated cases, RRs increased with age from 0.43 (95% confidence interval: 0.25–0.73) for 0- to 14-year olds to 44.9 (33.5–60.0) for ≥85-year olds, compared with 15- to 44-year olds. Males had an incidence of 1.28 (1.12–1.45) times that of females. Compared with non-Hispanic whites, the incidence was 1.57 (1.18–1.20) times higher among non-Hispanic Asians, 1.49 (1.22–1.83) among non-Hispanic blacks, and 1.73 (1.15–2.62) among Hispanics. Among females of childbearing age, non-Hispanic Asian females had 2.72 (1.51–4.89) and Hispanic females 3.13 (2.12–4.89) times higher incidence than non-Hispanic whites. We observed a higher percentage of deaths among older patient groups compared with 15- to 44-year olds. This study is the first characterizing higher RRs for listeriosis in the United States among non-Hispanic blacks and Asians compared with non-Hispanic whites. This information for public health risk managers may spur further research to understand if differences in listeriosis rates relate to differences in consumption patterns of foods with higher contamination levels, food handling practices, comorbidities, immunodeficiencies, health care access, or other factors.

## Introduction

In the United States, *Listeria monocytogenes* is an important foodborne pathogen that contributes to an estimated 1600 foodborne illnesses, 1500 hospitalizations, and 260 deaths each year (Scallan *et al.*, 2011). Invasive infections caused by *L. monocytogenes* (listeriosis) disproportionally affect pregnant females and their newborns, older adults, and individuals with comorbidities (Painter and Slutsker, 2007; Goulet *et al.*, 2008, 2012). In Europe, listeriosis incidence has increased among males 75 years of age and older and females older than 25 years (EFSA, 2016). In contrast, in the United States, no significant changes have been observed in the overall listeriosis incidence rate for the period 2006–2016 (Crim *et al.*, 2014, 2015; Marder *et al.*, 2017); however, recent trends in incidence in U.S. subgroups have not been evaluated.

Using surveillance data on foodborne illnesses reported by 10 state health departments in the United States to the Foodborne Diseases Active Surveillance Network (FoodNet), we examined the trends in listeriosis incidence by age, sex, race/ethnicity, and pregnancy status from 2008 to 2016. We also characterize the demographics of listeriosis incidence and mortality in the catchment areas and evaluate changes over the past decade in selected populations at an increased risk for listeriosis. Our study follows the work of Pouillot *et al.* (2012), which identified significantly higher incidence rate ratios (RRs) in the U.S. Hispanic population compared with non-Hispanic, and expands the analysis to include examination of the black and Asian populations.

## Materials and Methods

### FoodNet case data and population data

FoodNet is a collaboration among the Centers for Disease Control and Prevention (CDC), 10 state health departments, the U.S. Department of Agriculture's Food Safety and Inspection Service (USDA-FSIS), and the Food and Drug Administration (FDA), which conducts active, population-based surveillance for laboratory-confirmed infections caused by nine major pathogens transmitted commonly through food. FoodNet conducts surveillance for cases of listeriosis (laboratory confirmed by culture; illnesses diagnosed by culture-independent methods were confirmed by culture) reported in residents of Connecticut, Georgia, Maryland, Minnesota, New Mexico, Oregon, Tennessee, and selected counties in California, Colorado, and New York. Laboratories are audited to ensure full ascertainment of cases.

The FoodNet population covers 15% of the U.S. population. The FoodNet catchment population is thought to be demographically representative of the entire U.S. population, except for a lower percentage of persons of Hispanic ethnicity (CDC, 2011). In FoodNet, a case of invasive listeriosis is defined as isolation of *L. monocytogenes* from a normally sterile site (e.g., blood or cerebrospinal fluid or, less commonly, joint, pleural, or pericardial fluid) or from products of conception (e.g., placental or fetal tissue) in the setting of miscarriage or stillbirth. Culture-independent diagnostic tests (CIDT) were added as a criterion for case inclusion in 2012, but no CIDT positive case was in the database for 2012–2016.

We included all identified cases from January 1, 2008, to December 31, 2016, in residents of a FoodNet catchment area. Since 2004, FoodNet surveillance for laboratory-confirmed cases has been conducted continuously in the same geographically defined catchment area. Consistent with exclusions applied by previous researchers (Pouillot *et al.*, 2012; Silk *et al.*, 2012), we excluded cases designated as outbreak associated (<7% of all cases) to describe the incidence of sporadic listeriosis. Case data for the year of *L. monocytogenes* isolation, state of residence, sex, race/ethnicity, and age were evaluated as covariates in the analyses.

Population denominator data were obtained from the U.S. Census Bureau's annual population estimates for the FoodNet catchment areas during the 2008–2016 period (see also Supplementary Data).

### Data transformation

For analysis, we combined race and ethnicity into exclusive categories for the “Hispanic,” “non-Hispanic black,” “non-Hispanic white,” and “Asian,” which includes non-Hispanic Asian and Pacific Islander populations. The “Hispanic” category includes persons of any race identifying as being of Hispanic ethnicity. Other non-Hispanic individuals (American Indian, Alaska Native, and individuals who identified more than one race) were categorized as “non-Hispanic Other.” In preliminary analyses, we found 9% of race/ethnicity records were missing. To increase statistical power, minimize potential biases, and allow for inclusion of all race/ethnicity categories, we imputed missing observations as part of our model using the method described in the Supplementary Data.

Year of *L. monocytogenes* isolation was grouped into 3-year periods: 2008–2010, 2011–2013, or 2014–2016, and age was grouped into the following intervals: >31 d to 14 years, 15–44, 45–59, 60–69, 70–79, 80–84, and ≥85 years. These age groups were chosen to match those used by Pouillot *et al.* (2012) to follow trends over time.

Pregnancy-associated cases were defined as isolation of *L. monocytogenes* from a pregnant female, fetus, products of conception, or infant ≤31 d old. Documented mother-infant pairs (isolates obtained from both mother and infant) were counted as single pregnancy-associated cases and were attributed to the mother. Isolates obtained from fetuses or infants ≤31 d old without a corresponding maternal isolate were attributed to a pregnant female 15–44 years of age during the same year, state, and race/ethnicity because the demographic characteristics of the mother were unknown.

### Statistical analyses

All statistical analyses were performed in R version 3.4.2 (R Development Core Team, 2008).

### Incidence rates

To identify significant changes in case occurrence over time, incidence rates were estimated using negative binomial regression, which accounts for overdispersion, as described by Henao *et al.* (2010) (Table 1). Incidence rates from 2011 to 2013 and 2014 to 2016 were compared with the 2008–2010 period.

**Table 1.** Estimated Listeriosis Incidence Rates (per 100,000) and Rate Ratio (Reference Period: 2008–2010 Within Each Populations) in the FoodNet Catchment Area 2008–2016, Using a Negative Binomial Regression Model per Henao *et al*.

*Cases considered*	*All cases (general population),*^a^*95% CI*	*Nonpregnancy-associated cases,*^b^*95% CI*	*70+ years of age cases, 95% CI*	*Pregnancy-associated cases,*^c^*95% CI*
2008–2010	0.31 (0.24–0.42) (Ref.)	0.27 (0.20–0.36) (Ref.)	1.60 (1.17–2.24) (Ref.)	4.92 (3.35–7.32) (Ref.)
2011–2013	0.26 (0.20–0.35)	0.24 (0.18–0.33)	1.18 (0.85–1.66)	2.59 (1.62–4.10)
	RR: 0.84 (0.56–1.26); *p* = 0.40	RR: 0.90 (0.59–1.37); *p* = 0.62	RR: 0.73 (0.46–1.17); *p* = 0.19	RR: 0.53 (0.29–0.96); *p* = 0.04
2014–2016	0.26 (0.20–0.35)	0.23 (0.17–0.31)	1.21 (0.88–1.70)	3.64 (2.38–5.57)
	RR: 0.84 (0.56–1.26); *p* = 0.39	RR: 0.86 (0.56–1.30); *p* = 0.47	RR: 0.76 (0.48–1.20); *p* = 0.23	RR: 0.74 (0.41–1.31); *p* = 0.30

^a^ The denominator is the population >31 d old.

^b^ The denominator is the nonpregnant population >31 d old.

^c^ The denominator is the pregnant population.

95% CI, 95% confidence interval; FoodNet, Foodborne Diseases Active Surveillance Network; RR, rate ratio.

Source: Henao *et al.* (2010).

To explore and illustrate the relationship between age and the incidence of listeriosis, we constructed independent nonparametric Poisson regression models stratified by sex and race/ethnicity (Hispanic, non-Hispanic white, and non-Hispanic black) (Bowman and Azzalini, 1997). This method estimates a regression curve using a local likelihood approach for a vector of binomial observations, with a smoothing parameter set to 4 years.

### Rate ratios

To compare rates among different populations, negative binomial models were used to calculate adjusted RRs for the covariates. State of residence was included in every model to control for variation in incidence by geographic area (Henao *et al.*, 2010). Interaction was assessed with the relative excess risk due to interaction and the corresponding 95% confidence intervals (95% CI) (VanderWeele and Knol, 2014). Interactions were included if significant at the 0.05 level after consideration for multiple testing. Confounding variables were included if their presence resulted in ≥10% change in the coefficients of interest.

Separate models were constructed for females of childbearing age (defined as females 15–44 years of age) and nonpregnancy-associated cases, which included males ≥31 d of age and nonpregnant females ≥31 d of age. The “non-Hispanic Other” category for race/ethnicity was removed from the model for females of childbearing age as no case was observed in this population.

### Mortality rate

FoodNet categorizes the patient's outcome as “dead” or “alive” within 7 d of specimen collection date for nonhospitalized cases and at discharge for hospitalized cases. This represents all-cause mortality for patients with a diagnosis of listeriosis and does not confirm that the death is due to the illness. Three cases for which the outcome was unknown were excluded. Crude mortality rate by age, sex, period, and race/ethnicity with binomial CIs were evaluated for nonpregnancy-associated cases. Descriptive mortality data for the mother-infant pairs are provided.

## Results

A total of 1122 cases of nonoutbreak-associated invasive listeriosis recorded in the FoodNet catchment area from 2008 to 2016 met our criteria of inclusion; 153 (14%) were pregnancy-associated cases and 969 (86%) were nonpregnancy-associated cases. Estimated crude listeriosis incidence rates are available in Supplementary Table 1 and the numbers of listeriosis cases and person-year in each category in Supplementary Table 2.

**Table 2.** Listeriosis Rate Ratio in the FoodNet Catchment Area 2008–2016 by Age, Sex, Period, and Race/Ethnicity for the Nonpregnant Population

*Variable*	*Categories*^a^	*Cases*	*Rate ratio*	*95% CI*
Age (years)	0–14^b^	16	0.43	0.25–0.73
	15–44	78	Reference	NA
	45–59	163	4.14	3.14–5.44
	60–69	225	12.40	9.49–16.2
	70–79	236	24.50	18.8–32.0
	80–84	110	35.80	26.5–48.4
	85+	141	44.90	33.5–60.0
Sex	Female	481	Reference	NA
	Male	488	1.28	1.12–1.45
Period (years)	2008–2010	343	Reference	NA
	2011–2013	314	0.86	0.73–1.00
	2014–2016	312	0.80	0.68–0.93
Race/ethnicity^c^	Non-Hispanic white	617	Reference	NA
	Hispanic	76	1.73	1.15–2.62
	Non-Hispanic black	121	1.49	1.22–1.83
	Non-Hispanic Asian	62	1.57	1.18–2.10
	Non-Hispanic Other	9	0.97	0.41–2.28

^a^ The model is adjusted for state of residence.

^b^ The youngest age group is 32 d to 14 years of age and does not include infants ≤31 d old.

^c^ The sum of the cases listed by race/ethnicity category in the modeled results does not equal the total number of cases seen in the data as the model imputes race/ethnicity in 84 cases (thus adding 84 cases where the race/ethnicity is “known” in the model, while this was unknown in the raw data).

### Incidence rates

The annual invasive listeriosis incidence rate for 2008–2016 was 0.28 cases per 100,000 for the general population (excluding pregnant females), and among pregnant females, it was 3.73 cases per 100,000 pregnant females. Among adults ≥70 years of age, the annual incidence rate was 1.33 cases per 100,000 population. Of the periods compared during 2008–2016, there was no significant overall change in the estimated listeriosis incidence rate, except for a small, but significantly lower pregnancy-associated incidence rate in 2011–2013 compared with 2008–2010 (Table 1).

**FIG. 1.**
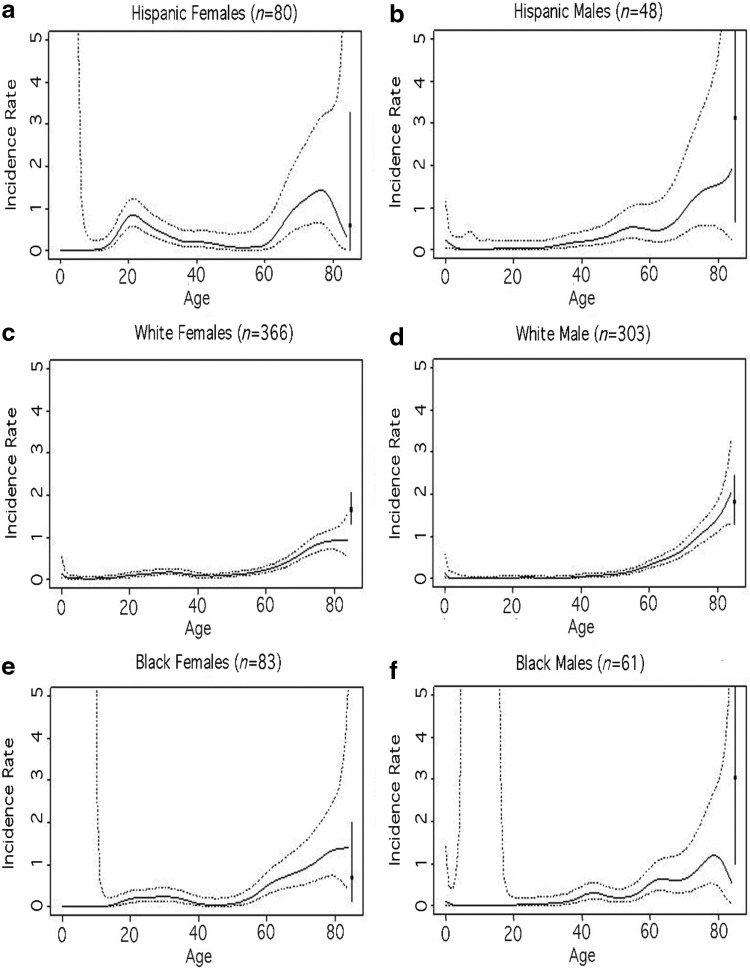
Incidence rate of listeriosis (per 100,000 population) by age (range 0–84 years): **(a)** Hispanic females, **(b)** Hispanic males, **(c)** non-Hispanic white females, **(d)** non-Hispanic white males, **(e)** non-Hispanic black females, and **(f)** non-Hispanic black males, in the FoodNet Catchment Area 2008–2016, as estimated by nonparametric logistic regression (Bowman and Azzalini, 1997). Continuous lines represent estimates; dashed lines, 95% CI bands. Black squares represent estimates for persons >85 years of age and the 95% Poisson CI. The youngest age group is 32 d to 14 years of age and does not include infants ≤31 d old. CI, confidence interval; FoodNet, Foodborne Diseases Active Surveillance Network.

Among females, incidence shows a bump during the childbearing years, and the higher incidence was most pronounced among Hispanic females (Fig. 1). Among males, incidence slightly increased around age 45 years for non-Hispanic black males, around 50 years for Hispanic males, and around 55 years for non-Hispanic white males. Both males and females of all race/ethnicities have a dramatic increase in incidence among older persons (≥70 years).

### RRs in nonpregnancy-associated cases

The model was adjusted for period, state of residence, sex, age group, and race/ethnicity; no other confounders or interaction terms were identified for inclusion (Table 2). Among the nonpregnant population, the RRs increased with age from 0.43 (95% CI: 0.25–0.73) for the 0- to 14-year olds to 44.9 (33.5–60.0) for the 85±-year olds, all compared with the 15- to 44-year olds. Males had an incidence of 1.28 (1.12–1.45) times that of females. Compared with non-Hispanic whites, non-Hispanic Asians, non-Hispanic blacks, and Hispanics had significantly higher incidences.

### RRs in females of childbearing age

The final model included the variables year, race/ethnicity, and pregnancy status; no confounders or interaction terms were identified for inclusion (Table 3). Among females of childbearing age, non-Hispanic Asians (RR: 2.72 [95% CI: 1.51–4.89]) and Hispanics (3.13 [2.12–4.62]) had significantly higher incidences compared with non-Hispanic whites.

**Table 3.** Listeriosis Rate Ratio in the FoodNet Catchment Area 2008–2016 by Year, Race/Ethnicity, and Pregnancy Status for Females of Childbearing Age (15–44 Years of Age)

*Variable*	*Categories*^a^	*Cases*	*Rate ratio*	*95% CI*
Year	2008–2010	81	Reference	NA
	2011–2013	45	0.58	0.40–0.85
	2014–2016	59	0.76	0.53–1.10
Race/ethnicity^b^	Non-Hispanic white	63	Reference	NA
	Hispanic	59	3.13	2.12–4.62
	Non-Hispanic black	28	1.54	0.97–2.44
	Non-Hispanic Asian	13	2.72	1.51–4.89
Pregnancy status	No	32	Reference	NA
	Yes	153	91.00	61.5–135

^a^ Model adjusted for state of residence. The race/ethnicity “Non-Hispanic Other” was removed from the analysis.

^b^ The sum of the cases listed by Race/Ethnicity category in the modeled results does not equal the total number of cases seen in the data as the model imputes Race/ethnicity in 22 cases (thus adding 22 cases where the race/ethnicity is “known” in the model, while this is unknown in the raw data).

### Mortality rates

A higher percentage of deaths were seen among the older age groups, compared with the 15- to 44-year age group (Table 4). Among 153 pregnancy-associated cases, 42 of the 122 with outcome data available (34%) reported death of the fetus or infant.

**Table 4.** Mortality Observed for Listeriosis Cases in the FoodNet Catchment Area 2008–2016 by Age, Sex, Period, and Race/Ethnicity for the Nonpregnancy-Associated Cases (*N* = 966)

*Variable*	*Categories*	*Cases*	*Deaths*	*% (95% CI)*
Age (years)	0–14^a^	16	1	6.2 (0.2–30.2)
	15–44	78	7	9.0 (3.7–17.6)
	45–59	163	27	16.6 (11.2–23.2)
	60–69	224^b^	32	14.3 (10.0–19.6)
	70–79	235^b^	36	15.3 (11.0–20.6)
	80–84	110	22	20.0 (13.0–28.7)
	85+	140^b^	33	23.6 (16.8–31.5)
Sex	Female	480^b^	74	15.4 (12.3–19.0)
	Male	486^c^	84	17.3 (14.0, 20.9)
Period (years)	2008–2010	343	57	16.6 (12.8–21.0)
	2011–2013	313^b^	57	18.2 (14.1–22.9)
	2014–2016	310^c^	44	14.2 (10.5–18.6)
Race/Ethnicity	Non-Hispanic white	615^c,d^	107	17.4 (14.5–20.6)
	Hispanic	76	11	14.5 (7.5–24.4)
	Non-Hispanic black	121	16	13.2 (7.8–20.6)
	Non-Hispanic Asian	62	12	19.4 (10.4–31.4)
	Non-Hispanic Other	9	1	11.1 (0.3–48.2)

^a^ The youngest age group is 32 d to 14 years of age and does not include infants ≤31 d old.

^b^ One missing data for the outcome (death/alive).

^c^ Two missing data for the outcome (death/alive).

^d^ Eighty-three missing data for the race/ethnicity.

## Discussion

To our knowledge, this study is the first reporting higher rates of listeriosis in the non-Hispanic black and Asian populations compared with the non-Hispanic white population. Among females of childbearing age (15–44 years), we identified race/ethnicity to be a significant risk factor for listeriosis with non-Hispanic Asian and Hispanic females having higher rates compared with non-Hispanic white females. Non-Hispanic black females of childbearing age (15–44 years) were not at significantly increased risk compared with non-Hispanic white females.

We found that among females of childbearing age (15–44 years), pregnant females had a significantly higher incidence of listeriosis with an RR of 91.0 (CI: 61.5–135) compared with nonpregnant females in this age group. The incidence in pregnancy-associated cases decreased significantly in the 2011–2013 period compared with the 2008–2010 period; although the incidence was also lower in the 2014–2016 period compared with the 2008–2010 period, the difference was not significant and it is unclear whether this decrease represents a longer-term trend.

In addition, our study found that U.S. males, older adults, and pregnant females have higher incidences of listeriosis comparable to those previously estimated (Pouillot *et al.*, 2012; Silk *et al.*, 2012). Although the FoodNet population is thought to be generally representative of the U.S. population, the U.S. Hispanic population is undersampled within the catchment area, and therefore, this may influence the findings pertaining to the Hispanic population (CDC, 2011).

With respect to the overall nonpregnant population, our analysis shows no significant change in listeriosis incidence over the three periods. Our findings differ from recently published European incidence rates, which showed an increasing rate of listeriosis among males older than 75 years and females 25 years of age or older for the 2008–2015 period (EFSA, 2016). In contrast to the FoodNet 2008–2016 findings, the preliminary 2017 FoodNet data, which was not included in this study, show a 26% increase in listeriosis cases dispersed across the population compared with the 2014–2016 period (Marder *et al.*, 2018). As this is the first year showing an increase and the data are not finalized, this change will require further study.

With respect to our subgroup analyses, Pouillot *et al.* (2012) found U.S. Hispanics to be at significantly higher risk compared with non-Hispanics (RR: 1.8, CI: 1.3–2.5) using FoodNet data from 2004 to 2009, but evaluated the effect of Hispanic ethnicity only, not race (Pouillot *et al.*, 2012; Silk *et al.*, 2012). In addition, Pouillot *et al.* (2012) found that pregnant females had increased incidence (RR: 114.6, CI: 68.9–205.1) compared with females of childbearing age and Hispanic females had an independently elevated incidence (RR: 1.9) compared with non-Hispanic females, but that study did not evaluate race. Where comparable, the incidence RRs in our study for the Hispanic populations are not significantly different from those found by Pouillot *et al.* (2012).

Census data projections indicate increases in population sizes and proportions for both older adults and Hispanics over time (Census Bureau, 2014). Pohl *et al.* (2017) examined the effects of changing U.S. population demographics on listeriosis incidence rates in the United States and found that if exposure and infectivity were constant, U.S. listeriosis rates would be expected to increase from 0.25 in 2010 to 0.32 per 100,000 in 2030. The calculations showed that achieving a steady state in listeriosis incidence from 2010 to 2020 required a 12% reduction in exposure to *L. monocytogenes* across the entire population or a larger reduction in a specific population, such as among older adults or the immunocompromised, assuming unchanged infectivity (Pohl *et al.*, 2017).

An increase in the U.S. incidence rate was not observed from 2008 to 2016, suggesting a reduction in exposure (Pohl *et al.*, 2017). Results from the recent multiyear interagency Market Basket Survey, examining 27,389 ready-to-eat foods in the United States, also provide supportive evidence of reduction in exposure to foods associated commonly with *L. monocytogenes* contamination for the five food categories where comparison was possible (Luchansky *et al.*, 2017).

The significant decrease we observed in the rate of listeriosis among the nonpregnant population in 2014–2016 versus 2008–2010 and among pregnant females in 2011–2013 versus 2008–2010 after controlling for age, race, and sex and the lack of significant interaction between time and age, race, or sex is consistent with a diffuse reduction across the population rather than a more marked decrease in a single susceptible population (Pohl *et al.*, 2017).

Hispanic-type soft cheese has been implicated in numerous listeriosis outbreaks (Linnan *et al.*, 1988; MacDonald *et al.*, 2005; Cartwright *et al.*, 2013; Jackson *et al.*, 2018), due to lack of pasteurization or inadequate hygiene practices postpasteurization (Jackson *et al.*, 2018), and is suspected as a contributor to the increased risk of listeriosis among the Hispanic population (Silk *et al.*, 2012). Gillespie *et al.* (2010) found an association between listeriosis and “neighborhood deprivation.”

Whether the differences in listeriosis rates among the race/ethnicity groups are due to differing consumption patterns that include more foods with higher contamination levels, or differences in food handling practices, rates of comorbidities, care-seeking behaviors, or other undetermined causes is not currently known. The finding in the literature is inconclusive and this is an area where further research is needed. No suspected food source of *Listeria* has been identified that is more commonly consumed by the non-Hispanic black and Asian populations. Further studies on demographics, consumption patterns, and *L. monocytogenes* contamination in the food supply may shed light on the increased risk in these populations and on additional targeted prevention strategies.

We found that advanced age significantly increases the risk of death in listeriosis cases (all causes). Given that mortality, comorbidities, and immunodeficiencies naturally increase with age, this finding is not surprising, but more research into the underlying causes of this association could help disentangle the effects of confounding factors. Without more complete information on comorbidities among the cases, we were unable to evaluate their effect on mortality.

## Conclusions

This study provides information for public health risk managers. Our results show a relatively stable incidence rate of listeriosis in the United States among various subgroups for the period 2008–2016, despite active efforts to reduce *L. monocytogenes* contamination in the food supply. The findings suggest, however, that interventions targeted for specific susceptible subgroups may help reduce the risk of listeriosis as the demographics of the U.S. population continue to change.

## Supplementary Material

Supplemental data
