# Supplementary material for: Differences Among Incidence Rates of Invasive Listeriosis in the U.S. FoodNet Population by Age, Sex, Race/Ethnicity, and Pregnancy Status, 2008–2016
Source: Foodborne Pathog Dis. 2019 Apr 10;16(4):290–7. doi: 10.1089/fpd.2018.2548 (PMC6482898; doi:10.1089/fpd.2018.2548)
Supplement: Supplemental data [file Supp_Data.pdf]

Supplementary Data

Population at Risk

As we lacked information on the number of pregnant females in the catchment areas, we estimated it using a similar method to the one used in Silk *et al.* (2012) and Pouillot *et al.* (2012).

First, the number of pregnancies resulting in live birth by year, state, and race/ethnicity was estimated by taking 9/12th of the corresponding number of children <1 year of age for each year, state, and race/ethnicity combination.

We then added to these pregnancies the number of pregnancies not resulting in live birth. These were estimated using published, national, ethnicity-specific numbers of induced abortion and fetal loss extracted from Ventura *et al.* (2012; their table 3). As an example, for the Hispanic population, (Ventura *et al.*, 2012) reports 1,539,000 pregnancies for 1,041,000 live births in 2008, that is $(1539-1041)/1041=0.478$ pregnancies that did not result in live birth per one pregnancy that resulted in live birth. Similarly, using the data from Ventura *et al.* (2012; their table 3), we estimated this number to be 0.446 and 1.04 for the non-Hispanic white population and the non-Hispanic black population.

For the non-Hispanic Asian and non-Hispanic Other populations, we used the total number of pregnancies for all race/ethnicity groups (6,578,000 pregnancies) and subtracted the number of pregnancies for the Hispanic population, the non-Hispanic white population, and the non-Hispanic black population to obtain 445,000 pregnancies. Similarly, we obtained 288,000 live births, for an estimate of 0.545 pregnancies that did not result in live birth per one pregnancy that resulted in live birth in these Asian and Other Race/Ethnicity populations. Following the rationale of others (Jamieson *et al.*, 2009; Pouillot *et al.*, 2012; Silk *et al.*, 2012), 2/12th of the resulting estimates were used for these calculations assuming that the pregnancies that did not result in live birth lasted an average of 2 months.

Iterative model fitting and imputation of missing race/ethnicity observations

Step 1: Let $C(X)$ be the number of individuals with characteristics X as estimated by the U.S. Census Bureau. Given Y_{mis} the missing covariates and Y_{obs}, the observed covariates, define, $\Pr(Y_{\text{mis}})$ for each individual of state St, Year Y, age group A, and sex S with at least one missing value for race or ethnicity. Its prior probability of being of race $R=$[Asian, black, Indian, Mixed, white] and/or of ethnicity $E=$[Hispanic, non-Hispanic], is as follows:

$\Pr(R, E \mid St, Y, A, S)=C(R, E, St, Y, A, S)/C(St, Y, A, S)$ if race and ethnicity are missing or

$\Pr(R \mid St, Y, A, S, E)=C(R, E, St, Y, A, S)/C(St, Y, A, S, E)$ if race only is missing or

$\Pr(E \mid St, Y, A, S, R)=C(R, E, St, Y, A, S)/C(St, Y, A, S, R)$ if ethnicity only is missing.

Step 2: Assign to each individual with at least one missing value for race or ethnicity a temporary race/ethnicity, sampled using the prior probability $\Pr(Y_{\text{mis}})$ (binomial distribution if ethnicity is missing only, multinomial distribution otherwise);

Step 3: Fit a negative binomial model on the simulated dataset (Y_{obs} and Y_{mis}). Store the estimated coefficients in a matrix $\boldsymbol{Q}$ and their variance in a matrix $\boldsymbol{U}$.

Step 4: From the model, simulate a set of parameters for $\beta^{*}\sim N(Q,\boldsymbol{V})$ where Q is the vector of estimated coefficients and $\boldsymbol{V}$ is the corresponding variance, covariance matrix (one value per parameter), $r^{*}\sim N(\theta, V(\theta))$, where θ is the overdispersion parameter estimated from the negative binomial model and $V(\theta)$ is its variance, $\rho\sim gamma(r^{*}, r^{*})$ (one value per observation). One random set of expected probabilities of being infected is as follows:

$$Pr(\text{Inf}|Y_{mis})=\frac{\rho\ \exp((\ \beta^{*})\ Y_{mis}+\log(D))}{D}$$

where $\boldsymbol{D}$ is the population size. The posterior distribution of the probability of being of a given race or ethnicity is then proportional to $Pr(Y_{mis}|\text{Inf})\propto Pr(\text{Inf}|Y_{mis})Pr(Y_{mis})$ according to Bayes' theorem. Assign to each individual with at least one missing value for race or ethnicity a value, sampled using the posterior probability.

Steps 3 and 4 are repeated m times until convergence, following a burn-in phase in which estimates are discarded.

When convergence is reached, $\overline{Q}$ is estimated as the average of $\boldsymbol{Q}$. The between imputation variability is $B_m=\sum\left(Q-\overline{Q}\right)\left(Q-\overline{Q}\right)'/(m-1)$, the within imputation variability is $\overline{U}_m=\sum U_m/m$, and the associated variance-covariance of $\overline{Q}_m$ is $T_m=\overline{U}_m+\frac{m+1}{m}B_m$. Eventually, we estimate the confidence interval of Q using $Q_{inf}=\overline{Q}+T_m\times t(0.025, df)$ and $Q_{sup}=\overline{Q}+T_m\times t(0.975, df)$ with $df=(m-1)\left(1+\frac{\overline{U}_m}{B_m\left(1+\frac{1}{m}\right)}\right)^2$. (Rubin, 1996)

Crude incidence rates

The incidence rates and trends were also evaluated using crude rates, the ratio of the number of cases in each population divided by the corresponding number of person-years of the population. Crude incidence rates were compared using exact Poisson tests. The crude listeriosis incidence rate for 2008–2016 was 0.26 cases per 100,000 in the general population (1122/426,114,742), and the crude incidence rate among pregnant females was 3.73 cases per 100,000 pregnant females (153/4,111,816). Among adults ≥70 years of age, the mean crude incidence rate equaled 1.26 cases per 100,000 population (487/38,716,462).

Supplementary Table S1. Estimated Crude Listeriosis Incidence Rates (per 100,000) in the FoodNet Catchment Area 2008–2016

Cases considered (years)	*All cases (general population)*[a]	*Nonpregnancy-associated cases*[b]	*70+ Years of age cases*	*Pregnancy-associated cases*[c]
2008–2010	0.30 (0.27–0.33) (Ref.)	0.25 (0.22–0.27) (Ref.)	1.53 (1.32–1.77) (Ref.)	5.09 (3.98–6.42) (Ref.)
2011–2013	0.24 (0.22–0.27), $p < 0.01$	0.22 (0.20–0.25), NS; $p = 0.16$	1.10 (0.93–1.30), $p < 0.01$	2.51 (1.74–3.51), $p < 0.01$
2014–2016	0.25 (0.22–0.27), $p = 0.01$	0.21 (0.19–0.24), NS; $p = 0.08$	1.17 (0.99–1.36), $p = 0.01$	3.52 (2.60–4.67), NS; $p = 0.06$

[a]The denominator is the population >31 d old.
[b]The denominator is the nonpregnant population >31 d old.
[c]The denominator is the pregnant population.
FoodNet, Foodborne Diseases Active Surveillance Network.

Supplementary Table S2. Listeriosis Cases Seen in the FoodNet Catchment Area 2008–2016

Variable	*Categories*[a]	*No. of cases*	*Person-year*
Nonpregnancy-associated cases			
	All cases	969	426,114,742
Period	2008–2010	343	138,691,502
	2011–2013	314	142,213,845
	2014–2016	312	145,209,395
Age (years)	0–14	16	82,289,087
	15–44	78	170,617,342
	45–59	163	90,786,660
	60–69	225	43,705,191
	70–79	236	23,537,718
	80–84	110	7,473,735
	≥85	141	7,705,009
Sex	Female	481	215,058,461
	Male	488	211,056,281
Race/ethnicity	NH Asian	62	22,308,711
	NH Black	121	65,476,605
	Hispanic	76	48,728,898
	NH Other	9	11,605,095
	NH White	617	277,995,433
	Unknown[a]	84	NA
Pregnancy-associated cases			
	All cases	153	4,111,816
Period	2008–2010	71	1,394,298
	2011–2013	34	1,355,262
	2014–2016	48	1,362,256
Race/ethnicity	NH Asian	10	200,638
	NH Black	23	795,424
	Hispanic	52	778,508
	NH Other	0	243,894
	NH White	52	2,093,352
	Unknown[a]	16	NA

[a]Represents the number of cases with missing race/ethnicity data, these are the cases for which race/ethnicity is imputed in the models.
NH, non-Hispanic.

Supplementary References

Jamieson DJ, Honein MA, Rasmussen SA, et al. H1n1 2009 influenza virus infection during pregnancy in the USA. Lancet 2009;374:451–458.

Pouillot R, Hoelzer K, Jackson KA, et al. Relative risk of listeriosis in Foodborne Diseases Active Surviellance Network (FoodNet) sites according to age, pregnancy, and ethnicity. Clin Infect Dis 2012;54:S405–S410.

Rubin DB. Multiple imputation after 18+ years. Journal of the American Statistical Association 1996;91:473–489.

Silk BJ, Date KA, Jackson KA, et al. Invasive listeriosis in the Foodborne Diseases Active Surveillance Network (FoodNet), 2004–2009: Further targeted prevention needed for higher-risk groups. Clin Infect Dis 2012;54:S396–S404.

Ventura SJ, Curtin SC, Abma JC, Henshaw SK. Estimated pregnancy rates and rates of pregnancy outcomes for the United States, 1990-2008. National Vital Statistics Reports: from the Centers for Disease Control and Prevention, National Center for Health Statistics, National Vital Statistics System 2012;60:1–21.
